# Supplementary material for: Heat adaptation of phage T7 under an extended genetic code
Source: Virus Evol. 2021 Dec 1;7(2):veab100. doi: 10.1093/ve/veab100 (PMC8923235; doi:10.1093/ve/veab100)
Supplement: veab100_Supp [file veab100_supp.zip › Sequencing_Analysis.zip!/Sequencing_Analysis/S3/evidence/SNP_7.html]

BRESEQ :: Evidence


| Predicted mutation | | | | | | |
| --- | --- | --- | --- | --- | --- | --- |
| evidence | seq id | position | mutation | annotation | gene | description |
| RA | NC\_001604.1 | 21,285 | A→C | intergenic (–/–) | – / – | –/– |

| Read alignment evidence... | | | | | | | | | | | |
| --- | --- | --- | --- | --- | --- | --- | --- | --- | --- | --- | --- |
|  | seq id | position | | ref | new | freq | score (cons/poly) | reads | annotation | genes | product |
| \* | NC\_001604.1 | 21,285 | 0 | A | C | 88.1% | 4454.4 / 288.8 | 1762 | intergenic (–/–) | *–/–* | –/– |
| Reads supporting (aligned to +/- strand):  **ref** base A (106/102);  **new** base C (764/779);  **total** (875/887) | | | | | | | | | | | |
| Fisher's exact test for biased strand distribution *p*-value = 7.12e-01 | | | | | | | | | | | |
| Kolmogorov-Smirnov test that lower quality scores support variant *p*-value = 2.70e-01 | | | | | | | | | | | |

Only 100 of 1763 total aligned reads displayed.  

`GCCCATACATCCCGATTCGGATGGTCAGACTAGATGGTGAATCCTACGGTCGTTCGTACATTGAGGAATACTTAGGTGACTTACGGTCCCTTGAAAATCTCCAAGAGGCTATCGTCAAGATGTCCATGATTAGCTCTAAGGTTATCGGCTTAGTGAATCCTGCTGGTATCACCCAGCCACGCCGACTGACCAAAGCTCAGACTGGTGACTTCGTTACTGGTCGTCCAGAAGACATCTCGTTCCTCCAACTGGAGAAGCAAGCAGACTTTACTGTAGCTAAAGCCGTAAGTGACGCTATCGAGGCTCGCCTTTCGTTTGCCTTTATGTTGAACTCTGCGGTTCAGCGTACAGGTGAACGTGTGACCGCCGAAGAGATTCGGTATGTAGCTTCTGAACTTGAAGATACTTTAGGTGG‑TGTCTACTCTATCCTTTCTCAAGAATTACAATTGCCTCTGGTACGAGTGCTCTTGAAGCAACTACAAGCCACGCAACAGATTCCTGAGTTACCTAAGGAAGCCGTAGAGCCAACCATTAGTACAGGTCTGGAAGCAATTGGTCGAGGACAAGACCTTGATAAGCTGGAGCGGTGTGTCACTG  >  NC_001604.1/20985‑21581`  

                                                                                                                                                                                                                                                                                                            `|`                                                                                                                                                                                                                                                                                                           
             `gATTCGGATTGTGAGAATAGGTGGTGAATCCTACGGTCGTTCGTACATTGAGGAATACTTAGGTGACTTACGGTCCCTTGAAAATCTCCAAGAGGCTATCGTCAAGATGTCCATGATTAGCTCTAAGGTTATCGGCTTAGTGAATCCTGCTGGTATCACCCAGCCACGCCGACTGACCAAAGCTCAGACTGGTGACTTCGTTACTGGTCGTCCAGAAGACATCTCGTTCCTCCAACTGGAGAAGCAAGCAGACTTTACTGTAGCTAACGCCGTAAGTGACGCTATCGCGGCTCGCCTTTCg                                                                                                                                                                                                                                                                                              <  1:182194/301‑1 (MQ=255)`  
                 `cGGATGGTCAGACTAGATGGTGAATCCTACGGTCGTTCGTACATTGAGGAATACTTAGGTGACTTACGGTCCCTTGAAAATCTCCAAGAGGCTATCGTCAAGATGTCCATGATTAGCTCTAAGGTTATCGGCTTAGTGAATCCTGCTGGTATCACCCAGCCACGCCGACTGACCAAAGCTCAGACTGGTGACTTCGTTACTGGTCGTCCAGAATACATCTCGTTCCTCCAACTGGAGAAGCAAACAGACTTTACTGTAGCTAACGCCGTAAGTTATCTCATCGCGGCTCCCCTTTCGtttt                                                                                                                                                                                                                                                                                          >  1:331529/1‑300 (MQ=255)`  
                      `ggTCAGACTAGATGGTGAATCCTACGGTCGTTCGTACATTGAGGAATACTTAGGTGACTTACGGTCCCTTGAAAATCTCCAAGAGGCTATCGTCAAGATGTCCATGATTAGCTCTAAGGTTATCGGCTTAGTGAATCCTGCTGGTATCACCCAGCCACGCCGACTGACCAAAGCTCAGACTGGTGACTTCGTTACTGGTCGTCCAGAAGACATCTCGTTCCTCCAACTGGAGAAGCAAGCAGACTTTACTGTAGCTAACGCCGTAAGTGACGCTATCGCGGCTCGCCTTTCGTTTGCCttt                                                                                                                                                                                                                                                                                     >  1:25218/1‑301 (MQ=255)`  
                          `aGACTAGATGGTGAATCCTACGGTCGTTCGTACATTGAGGAATACTTAGGTGACTTACGGTCCCTTGAAAACCTCCAAGAGGCTATCGTCAAGATGTCCATGATTAGCTCTAAGGTTATCGGCTTAGTGAATCCTGCTGGTATCACCCAGCCACGCCGACTGACCAAAGCTCAGACTGGTGACTTCGTTACTGGTCGTCCAGAAGACATCTCGTTCCTCCAACTGGAGAAGCAAGCAGACTTTACTGTAGCTAACGCCGTAAGTGACGCTATCGAGGCTCGCCTTTCGTTTGCCTTTATGt                                                                                                                                                                                                                                                                                 <  1:291608/301‑1 (MQ=255)`  
                           `gACTAGATGGTGAATCCTACGGTCGTTCGTACATTGAGGAATACTTAGGTGACTTACGGTCCCTTGAAAATCTCCAAGAGGCTATCGTCAAGATGTCCATGATTAGCTCTAAGGTTATCGGCTTAGTGAATCCTGCTGGTATCACCCAGCCACGCCGACTGACCAAAGCTCAGACTGGTGACTTCGTTACTGGTCGTCCAGAAGACATCTCGTTCCTCCTACTGGAGAAGCTAGCAGACTTTACTGTAGCTAACGCCGTAAGTGACGCTATCGCGGCTCGCCTTTCGTTTGCCTTTATGtt                                                                                                                                                                                                                                                                                >  1:225662/1‑301 (MQ=255)`  
                              `tAGATGGTGAATCCTACGGTCGTTCGTACATTGAGGAATACTTAGGTGACTTACGGTCCCTTGAAAATCTCCAAGAGGCTATCGTCAAGATGTCCATGATTAGCTCTAAGGTTATCGG‑TTAGTGAATCCTGCTGGTATCACCCAGCCACGCCGACTGACCAAAGCTCAGACTGGTGACTTCGTTACTGGTCGTCCAGAAGACATCTCGTTCCTCCAACTGGAGAAGCAAGCAGACTTTACTGTAGCTAACGCCGTAAGTGACGCTATCGCGGCTCGCCTTTCGTTTGCCTTTATGTTGAAc                                                                                                                                                                                                                                                                            <  1:78952/301‑1 (MQ=255)`  
                               `aGATGGTGAATCCTACGGTCGTTCGTACATTGAGGAATACTTAGGTGACTTACGGTCCCTTGAAAATCTCCAAGAGGCTATCGTCAAGATGTCCATGATTAGCTCTAAGGTTATCGGCTTAGTGAATCCTGCTGGTATCACCCAGCCACGCCGACTGACCAAAGCTCAGACTGGTGACTTCGTTACTGGTCGTCCAGAAGACATCTCGTTCCTCCAACTGGAGAAGCAAGCAGACTTTACTGTAGCTAACGCCGTAAGTGACGCTATCGAGGCTCGCATTTCGTTTGCCTTTATGTTGAAc                                                                                                                                                                                                                                                                            <  1:142592/301‑1 (MQ=255)`  
                                   `ggTGAATCCTACGGTCGTTCGTACATTGAGGAATACTTAGGTGACTTACGGTCCCTTGAAAATCTCCAAGAGGCTATCGTCAAGATGTCCATGATTAGCTCTAAGGTTATCGGCTTAGTGAATCCTGCTGGTATCACCCAGCCACGCCGACTGACCAAAGCTCAGACTGGTGACTTCGTTACTGGTCGTCCAGAAGACATCTCGTTCCTCCAACTGGAGAAGCAAGCAGACTTTACTGTAGCTAACGCCGTAAGTGCCGCTATCGCGGCTCGCCTTTCGTTTGCCTTTATGTTGAACTCTg                                                                                                                                                                                                                                                                        >  1:342509/1‑301 (MQ=255)`  
                                      `gAATCCTACGGTCGTTCGTACATTGAGGAATACTTAGGTGACTTACGGTCCCTTGAAAATCTCCAAGAGGCTATCGTCAAGATGTCCATGATTAGCTCTAAGGTTATCGGCTTAGTGAATCCTGCTGGTATCACCCAGCCACGCCGACTGACCAAAGCTCAGACTGGTGACTTCGTTACTGGTCGTCCAGAAGACATCTCGTTCCTCCAACTGGAGAAGCAAGCAGACTTTACTGTAGCTAACGCCGTAAGTGACGCTATCGAGGCTCGCCTTTCGTTTGCCTTTATGTTGAACTCTGCgg                                                                                                                                                                                                                                                                     <  1:139453/301‑1 (MQ=255)`  
                                        `aTCCTACGGTCGTTCGTACATTGAGGAATACTTAGGTGACTTACGGTCCCTTGAAAATCTCCAAGAGGCTATCGTCAAGATGTCCATGATTAGCTCTAAGGTTATCGGCTTAGTGAATCCTGCTGGTATCACCCAGCCACGCCGACTGACCAAAGCTCAGACTGGTGACTTCGTTACTGGTCGTCCAGAAGACATCTCGTTCCTCCAACTGGAGAAGCAAGCAGACTTTACTGTAGCTAACGCCGTAAGTGACGCTATCGCGGCTCGCCTTTCGTTTGCCTTTATGTTGAACTCTGCGGtt                                                                                                                                                                                                                                                                   >  1:104508/1‑301 (MQ=255)`  
                                         `tCCTACGGTCGTTCGTACATTGAGGAATACTTAGGTGACTTACGGTCCCTTGAAAATCTCCAAGAGGCTATCGTCAAGATGTCCATGATTAGCTCTAAGGTTATCGGCTTAGTGAATCCTGCTGGTATCACCCAGCCACGCCGACTGACCAAAGCTCAGACTGGTGACTTCGTTACTGGTCGTCCAGAAGACATCTCGTTCCTCCGACTGGAGAAGCCAGCAGACTTTACTGTAGCTAACGCCGTAAGTGACGCTATCGCGGCTCGCCTTTCGTTTGCCTTTATGTTGAACTCTGCGGttt                                                                                                                                                                                                                                                                  >  1:288179/1‑300 (MQ=255)`  
                                         `tCCTACGGTCGTTCGTACAGTGAGGAATACTTAGGTGACTTACGGTCCCTTGAAAATCTCCAAGGGGCTATCGTCAAGATGTCCATGATTAGCTCTAAGGTTATCGGCTTAGTGAATCCTGCTGGTATCGCCCAGCCACGCCGACTGACCAAAGCTCAGACTGGTGACTTCGTTACTGGTCGTCCAGAAGACATCTCGTTCCTCCAACTGGAGAAGCAAGCAGACTTTACTGTAGCTAACGCCGTAAGTGACGCTATCGCGGCTCGCCTTTCGTTTGCCTTTATGTTGAACTCTGCGGTTc                                                                                                                                                                                                                                                                  <  1:35495/301‑1 (MQ=255)`  
                                           `cTACGGTCGTTCGTACATTGAGGAATACTTAGGTGACTTACGGTCCCTTGAAAATCTCCAAGAGGCTATCGTCAAGATGTCCATGATTAGCTCTAAGGTTATCGGCTTAGTGAATCCTGCTGGTATCACCCAGCCACGCCGACTGACCAAAGCTCAGACTGGTGACTTCGTTACTGGTCGTCCAGAAGACATCTCGTTCCTCCAACTGGAGAAGCAAGCAGACTTTACTGTAGCTAACGCCGTAAGTGACGCTATCGCGGCTCGCCTTTCGTTTGCCTTTATGtctgtctcttatacacat                                                                                                                                                                                                                                                                >  1:309777/1‑284 (MQ=255)`  
                                              `cGGTCGTTCGTACATTGAGGAATACTTAGGTGACTTACGGTCCCTTGAAAATCTCCAAGAGGCTATCGTCAAGATGTCCATGATTAGCTCTAAGGTTATCGGCTTAGTGAATCCTGCTGGTATCACCCAGCCACGCCGACTGACCAAAGCTCAGACTGGTGACTTCGTTACTGGTCGTCCAGAAGACATCTCGTTCCTCCAACTGGAGAAGCAAGCAGACTTTACTGTAGCTAACGCCGTAAGTGACGCTATCGCGGCTCGCCTTTCGTTTGCCTTTATGTTGAACTCTGCGGTTCAGCGt                                                                                                                                                                                                                                                             <  1:17762/301‑1 (MQ=255)`  
                                                `gTCGTTCGTACATTGAGGAATACTTAGGTGACTTACGGTCCCTTGAAAATCTCCAAGAGGCTATCGTCAAGATGTCCATGATTAGCTCTAAGGTTATCGGCTTAGTGAATCCTGCTGGTATCACCCAGCCACGCCGACTGACCAAAGCTCAGACTGGTGACTTCGTTACTGGTCGTCCAGAAGACATCTCGTTCCTCCAACTGGAGAAGCAAGCAGACTTTACTGTAGCTAACGCCGTAAGTGACGCTATCGCGGCTCGCCTTTCGTTTGCCTTTATGTTGAACTCTGCGGTTCAGCGTAc                                                                                                                                                                                                                                                           <  1:119916/301‑1 (MQ=255)`  
                                                  `cgttcgtACATTGAGGAATACTTAGGTGACTTACGGTCCCTTGAAAATCTCCAAGAGGCTATCGTCAAGATGTCCATGATTAGCTCTAAGGTTATCGGCTTAGTGAATCCTGCTGGTATCACCCAGCCACGCCGACTGACCAAAGCTCAGACTGGTGACTTCGTTACTGGTCGTCCAGAAGACATCTCGTTCCTCCAACTGGAGAAGCAAGCAGACTTTACTGTAGCTAACGCCGTAAGTGACGCTATCGCGGCTCGCCTTTCGTTTGCCTTTATGTTGAACTCTGCGGTTCAGCGTACAg                                                                                                                                                                                                                                                         >  1:26290/1‑301 (MQ=255)`  
                                                   `gttcgtACATTGAGGAATACTTAGGTGACTTACGGTCCCTTGAAAATCTCCAAGAGGCTATCGTCAAGATGTCCATGATTAGCTCTAAGGTTATCGGCTTAGTGAATCCTGCTGGTATCACCCAGCCACGCCGACTGACCAAAGCTCAGACTGGTGACTTCGTTACTGGTCGTCCAGAAGACATCTCGTTCCTCCAACTGGAGAAGCAAGCAGACTTTACTGTAGCTAACGCCGTAAGTGACGCTATCGCGGCTCGCCTTTCGTTTGCCTTTATGTTGAACTCTGCGGTTCAGCGTACAgg                                                                                                                                                                                                                                                        <  1:51047/301‑1 (MQ=255)`  
                                                    `ttcgtACATTGAGGAATACTTAGGTGATTTACGGTCTCTTGAAAATCTCCAAGAGGCTATCGTCAAGATGTCCATGATTAGCTCTAAGGTTATCGGCTTAGTGAATCCTGCTGGTATCACCCAGCCACGCCGACTGACCAAAGCTCAGACTGGTGACTTCGTTACTGGGCGTACAGAAGACATCTCGTTCCTCCAACTGGAGAAGCAAGCAGACTTTACTGTAGCTCACGCCGTAAGTGACGCTATCGCGGCTCGCCTTTCGTTTTCCTTTATGTTCTACTCTGCGGTTCAGCGTACAGGt                                                                                                                                                                                                                                                       >  1:92484/1‑301 (MQ=255)`  
                                                    `ttcgtACATTGAGGAATACTTAGGTGACTTACGGTCCCTTGAAAATCTCCAAGAGGCTATCGTCAAGATGTCCATGATTAGCTCTAAGGTTATCGGCTTAGTGAATCCTGCTGGTATCACCCAGCCACGCCGACTGACCAAAGCTCAGACTGGTGACTTCGTTACTGGTCGTTCAGAAGACATCTCGTTCCTCCAACTGGAGAAGCAAGCAGACTTTACTGTAGCTAACGCCGTAAGTGACGCTATCTCGGCTCGCCTTTCGTTTGCCTTTATGTTGAACTCTGCGGTTCAGCGTACAGGt                                                                                                                                                                                                                                                       >  1:110788/1‑301 (MQ=255)`  
                                                    `ttcgtACATTGAGGAATACTTAGGTGACTTACGGTCCCTTGAAAATCTCCAAGAGGCTATCGTCAAGATGTCCATGATTAGCTCTAAGGTTATCGGCTTAGTGAATCCTGCTGGTATCACCCAGCCACGCCGACTGACCAAAGCTCAGACTGGTGACTTCGTTACCGGTCGTCCAGAAGACATCTCGTTCCTCCAACTGGAGAAGCAAGCAGACTTTACTGTAGCTAACGCCGTAAGTGACGCTATCGCGGCTCGCCTTTCGTTTGCCTTTATGTTGAACTCTTCGGTTAAGCGTACAGGt                                                                                                                                                                                                                                                       >  1:33798/1‑301 (MQ=255)`  
                                                     `ctcggagatgggtaaaagaaacaggACTTACGGTCCCTTGAAAATCTCCAAGAGGCTATCGTCAAGATGTCCATGATTAGCTCTAAGGTTATCGGCTTAGTGAATCCTGCTGGTATCACCCAGCCACGCCGGCTGACCAAAGCTCAGACTGGTGACTTCGTTACTGGTCGTCCAGAAGACATCTCGTTCCTCCAACTGGAGAAGCAAGCAGACTTTACTGTAGCTAACGCCGTAAGTGACGCTATCGCGGCTCGCCTTTCGTTTGCCTTTATGTTGAACTCTGCGGTTCAGCGTACAGGTg                                                                                                                                                                                                                                                      <  1:69550/277‑1 (MQ=255)`  
                                                          `aaTTGAGGAATACTTAGGTGACTTACGGTCCCTTGAAAATCTCCAAGAGGCTATCGTCAAGATGTCCATGATTAGCTCTAAGGTTATCGGCTTAGTGAATCCTGCTGGTATCACCCAGCCACGCCGACTGACCAAAGCTCAGACTGGTGACTTCGTTACTGGTCGTCCAGAAGACATCTCGTTCATCCAACTGGAGAAGCAAGCAGACTTTACTGTAGCTAACGCCGTAAGTGACGCTATCGCGGCTCGCCTTTCGTTTGCCTTTATGTTGAACTCTGCGGTTCAGCGTACAGGTGAACgt                                                                                                                                                                                                                                                 <  1:62238/300‑1 (MQ=255)`  
                                                           `aTTGAGGAATACTTAGGTGACTTACGGTCCCTTGAAAATCTCCAAGAGGCTATCGTCAAGATGTCCATGATTAGCTCTAAGGTTATCGGCTTAGTGAATCCTGCTGGTATCACCCAGCCACGCCGACTGACCAAAGCTCAGACTGGTGACTTCGTTACTGGTCGTCCAGAAGACATCTCGTTCCTCCAACTGGAGAAGCAAGCAGACTTTACTGTAGCTAACGCCGTAAGTGACGCTATCGCGGCTCGCCTTTCGTTTGCCTTTATGTTGAACTCTGCGGTTCAGCGTACAGGTGAACgtg                                                                                                                                                                                                                                                <  1:196921/301‑1 (MQ=255)`  
                                                                `gtataagagacaggACTTACGGTCCCTTGAAAATCTCCAAGAGGCTATCGTCAAGATGTCCATGATTAGCTCTAAGGTTATCGGCTTAGTGAATCCTGCTGGTATCACCCAGCCACGCCGACTGACCAAAGCTCAGACTGGTGACTTCGTTACTGGTCGTCCAGAAGACATCTCGTTCCTCCAACTGGAGAAGCAAGCAGACTTTACTGTAGCTAACGCCGTAAGTGACGCTATCGCGGCTCGCCTTTCGTTTGCCTTTATGTTGAACTCTGCGGTTCAGCGTACAGGTGAACGTGTGAcc                                                                                                                                                                                                                                           <  1:240866/288‑1 (MQ=255)`  
                                                                `ggAATACTTAGGTGACTTACGGTCCCTTGAAAATCTCCAAGAGGCTATCGTCAAGATGTCCATGATTAGCTCTAAGGTTATCGGCTTAGTGAATCCTGCTGGTATCACCCAGCCACGCCGACTGACCAAAGCTCAGACTGGTGACTTCGTTACTGGTCGTCCAGAAGACATCTCGTTCCTCCAACTGGAGAAGCAAGCAGACTTTACTGTAGCTAACTCCGTAAGTGACGCTATCTCGGCTCGCCTTTCGTTTGCCTTTATGTTGAACTCTGCGGTTCAGCGTACCTGTGAACGTTTGAcc                                                                                                                                                                                                                                           >  1:4381/1‑301 (MQ=255)`  
                                                                `ggAATACTTAGGTGACTTACGGTCCCTTGAAAATCTCCAAGAGGCTATCGTCAAGATGTCCATGATTAGCTCTAAGGTTATCGGCTTAGTGAATCCTGCTGGTATCACCCAGCAACGCCGACTGACCAAAGCTCAGACTGGTGACTTCGTTACTGGTCGTCCAGAAGACATCTCGTTCCTCCAACTGGAGAAGCAAGCAGACTTTACTGTAGCTAACGCCGTAAGTGACGCTATCGCGGCTCGCCTTTCGTTTGCCTTTATGTTGAACTCTGCGGTTCAGCGTACAGGTgctgtctcttat                                                                                                                                                                                                                                           >  1:56343/1‑290 (MQ=255)`  
                                                                  `aaTACTTAGGTGACTTACGGTCCCTTGAAAATCTCCAAGAGGCTATCGTCAAGATGTCCATGATTAGCTCTAAGGTTATCGGCTTAGTGAATCCTGCTGGTATCACCCAGCCACGCCGACTGACCAAAGCTCAGACTGGTGACTTCGTTACTGGTCGTCCAGAAGACATCTCGTTCCTCCAACTGGAGAAGCAAGCAGACTTTACTGTAGCTAACGCCGTAAGTGACGCTATCGCGGCTCGCCTTTCGTTTGCCTTTATGTTGAACTCTGCGGTTCAGCGTACAGGTGAACGTGTGAccgc                                                                                                                                                                                                                                         <  1:312743/301‑1 (MQ=255)`  
                                                                   `aTACTTAGGTGACTTACGGTCCCTTGAAAATCTCCAAGAGGCTATCGTCAAGATGTCCATGATTAGCTCTAAGGTTATCGGCTTAGTGAATCCTGCTGGTATCACCCAGCCACGCCGACTGACCAAAGCTCAGACTGGTGACTTCGTTACTGGTCGTCCAGAAGACATCTCGTTCCTCCAACTGGAGAAGCAAGCAGACTTTACTGTAGCTAACGCCGTAAGTGACGCTATCGCGGCTCGCCTTTCGTTTGCCTTTATGTTGAACTCTGCGGTTCAGCGTACAGGTGAACGTGTGAccgcc                                                                                                                                                                                                                                        >  1:107704/1‑301 (MQ=255)`  
                                                                      `cTTAGGTGACTTACGGTCCCTTGAAAATCTCCAAGAGGCTATCGTCAAGATGTCCATGATTAGCTCTAAGGTTATCGGCTTAGTGAATCCTGCTGGTATCACCCAGCCACGCCGACTGACCAAAGCTCAGACTGGTGACTTCGTTACTGGTCGTCCAGAAGACATCTCGTTCCTCCAACTGGAGAAGCAAGCAGACTTTACTGTAGCTAACGCCGTAAGTGACGCTATCGCGGCTCGCCTTTCGTTTGCCTTTATGTTGAACTCTGCGGTTCAGCGTACAGGTGAACGTGTGACCGCCGaa                                                                                                                                                                                                                                     >  1:281040/1‑301 (MQ=255)`  
                                                                          `ggTGACTTACGGTCCCTTGAAAATCTCCAAGAGGCTATCGTCAAGATGTCCATGATTAGCTCTAAGGTTATCGGCTTAGTGAATCCTGCTGGTATCACCCAGCCACGCCGACTGACCAAAGCTCAGACTGGTGACTTCGTTACTGGTCGTCCAGAAGACATCTCGTTCCTCCAACTGGAGAAGCAAGCAGACTTTACTGTAGCTAACGCCGTAAGTGACGCTATCGCGGCTCGCCTTTCGTTTGCCTTTATGTTTAACTCTGCGGTTCAGCGTACAGGTGAACGTGTGACCGCCGAAgaga                                                                                                                                                                                                                                 >  1:234493/1‑301 (MQ=255)`  
                                                                               `cTTACGGTCCCTTGAAAATCTCCAAGAGGCTATCGTCAAGATGTCCATGATTAGCTCTAAGGTTATCGGCTTAGTGAATCCTGCTGGTATCACCCAGCCACGCCGACTGACCAAAGCTCAGACTGGTGACTTCGTTACTGGTCGTCCAGAAGACATCTCGTTCCTCCAACTGGAGAAGCAAGCAGACTTTACTGTAGCTAACGCCGTAAGTGACGCTATCGCGGCTCGCCTTTCGTTTGCCTTTATGTTGAACTCTGCGGTTCAGCGTACAGGTGAACGTGTGACCGCCGAAGAGATTCgg                                                                                                                                                                                                                            >  1:266428/1‑301 (MQ=255)`  
                                                                                `gagatgtttataagagacagccAAGAGGCTATCGTCAAGATGTCCATGATTAGCTCTAAGGTTATCGGCTTAGTGAATCCTGCTGGTATCACCCAGCCACGCCGACTGACCAAAGCTCAGACTGGTGACTTCGTTACTGGTCGTCCATAAGACATCTCGTTCCTCCAACTGGAGAAGCAAGCAGACTTTACTGTAGCTAACGCCGTAAGTGACGCTATCGCGGCTCGCCTTTCGTTTGCCTTTATGTTGAACTCTGCGGTTCAGCGTACAGGTGAACGTGTGACCGCCGAAGAGATTCGGt                                                                                                                                                                                                                           <  1:184463/281‑1 (MQ=255)`  
                                                                                      `gcccTTGAAAAACTCCAAGAGGCCATCGTAAAGAAGGCCATGATTAGCTCGAAGGGTATCGGCTTAGGGAAACCTGCTGGTATCACCCAGCCACGCCGACTGACCAAAGCGCAGACTGGGGAATTCGGTACCGGTCGTCCAGAAGACATCTCGTTCCTCCAACTGGAGAAGCAAGCAGACTTTACTGTAGCTAACGCCGTAAGTGACGATATCGCGGCTCGCCTTTCGTTTGCCTTTATGTTGAACTCTGCGGTTCAGCGTGCAGGTGAACGTGTGACCGCCGAAGAGATTCGGTATGTAg                                                                                                                                                                                                                     <  1:223360/300‑1 (MQ=255)`  
                                                                                       `cccTTGAAAATCTCCAAGAGGCTATCGTCAAGATGTCCATGATTAGCTCTAAGGTTATCGGCTTAGTGAATCCTGCTGGTATCACCCAGCCACGCCGACTGACCAAAGCTCAGACTGGTGACTTCGTTACTGGTCGTCCAGAAGACATCTCGTTCCTCCAACTGGAGAAGCAAGCAGACTTTACTGTAGCTAACGCCGTAAGTGACGCTATCGCGGCTCGCCTTTCGTTTGCCTTTATGTTGAACTCTTCGGTTCAGCGTACAGGTGAACGTGTGACCGCCGAATAGATTCGGTATGTAGc                                                                                                                                                                                                                    >  1:20699/1‑301 (MQ=255)`  
                                                                                                       `agatgtgtataagagacagTCCATGATTAGCTCTAAGGTTATCGGCTTAGTGAATCCTGCTGGTATCACCCAGCCACGCCGACTGACCAAAGCTCAGACTGGTGACTTCGTTACTGGTCGTCCAGAAGACATCTCGTTCCTCCAACTGGAGAAGCAAGCAGACTTTACTGTAGCTAACGCCGTAAGTGACGCTATCGCGGCTCGCCTTTCGTTTGCCTTTATGTTGAACTCTGCGGTTCAGCGTACAGGTGAACGTGTGACCGCCGAAGAGATTCGGTATGTAGCTTCTGAACTTGAAGAt                                                                                                                                                                                                    <  1:72375/283‑1 (MQ=255)`  
                                                                                                          `ggCTATCGTCAAGATGTCCATGATTAGCTCTAAGGTTATCGGCTTAGTGAATCCTGCTGGTATCACCCAGCCACGCCGACTGACCAAAGCTCAGACTGGTGACTTCGTTACTGGTCGTCCAGAAGACATCTCGTTCCTCCAACTGGAGAAGCAAGCAGACTTTACTGTAGCTAACGCCGTAAGTGACGCTATCGCGGCTCGCCTTTCGTTTGCCTTTATGTTGAACTCTGCGGTTCAGCGTACAGGTGAACGTGTGACCGCCGAAGCGATTCGGTATGTAGCTTCTGAACTTGAAGATACt                                                                                                                                                                                                 >  1:259344/1‑301 (MQ=255)`  
                                                                                                              `aTCGTCAAGATGTCCATGATTAGCGCTAAGGTTATCGGCTTAGTGAATCCTGCTGGTATCACCCAGCCACGCCGACTGACCAAAGCTCAGACTGGTGACTTCGTTACTGGTCGTCCAGAAGACATCTCGTTCCTCCAACTGGAGAAGCAAGCAGACTTTACTGTAGCTAACGCCGTAAGTGACGCTATCGCGGCTCGCCTTTCGTTTGCCTTTATGTTGAACTCTGCGGTTCAGCGTACAGGTGAACGTGTGACCGCCGAAGAGATTCGGTATGTAGCTTCTGAACTTGAAGATACTTTAg                                                                                                                                                                                             <  1:315678/301‑1 (MQ=255)`  
                                                                                                              `aTCGTAAAGATGTCCATGATTAGCTCTAAGGTTATCGGCTTAGTGAATCCTGCTGGTATCACCCAGCCACGCCGACTGACCAAAGCTCAGACTGGTGACTTCGTTACTGGTCGTCCAGAAGACATCTCGTTCCTCCAACTGGAGAAGCAAGCAGACTTTACTGTAGCTAACGCCGTAAGTGACGCTATCGCGGCTCGCCTTTCGTTTGCCTTTATGTTGAACTCTGCGGTTCAGCGTACAGGTGAACGTGTGACCGCCGAAGAGATTCGGTATGTAGCTTCTGAACTTGAAGATACTTTAg                                                                                                                                                                                             <  1:191134/301‑1 (MQ=255)`  
                                                                                                               `tCGTCAAGATGTCCAGGATTAGCTCTAAGGTTATCGGCTTAGTGAATCCTGCGGGTATCACCCAGCCACGCCGACTGACCAAAGCTCAGACTGGTGACTTCGTTACTGGTCGTCCAGAAGAAATCTCGTTCCTCCAACTGGAGAAGCAAGCAGACTTTACTGTAGCTAACGCCGTAAGTGACGCTATCGCGGCTCGCCTTTCGGTTGCCTTTATGTTGAACTCTGCGGTTCAGCGTACAGGTGAACGTGTGACCGCCGAAGAGATTCGGTATGTAGCTTCTGAACTTGAAGATACTTTAgg                                                                                                                                                                                            <  1:161031/301‑1 (MQ=255)`  
                                                                                                                  `tCAAGATGTCCATGATTAGCTCTAAGGTTATCGGCTTAGTGAATCCTGCTGGTATCACCCAGCCACGCCGACTGACCAAAGCTCAGACTGGTGACTTCGTTACTGGTCGTCCAGAAGACATCTCGTTCCTCCAACTGGAGAAGCAAGCAGACTTTACTGTAGCTAACGCCGTAAGTGACGCTATCGCGGCTCGCCTTTCGTTTGCCTTTATGTTGAACTCTGCGGTTCAGCGTACAGGTGAACGTGTGACCGCCGAAGAGATTCGGTATGTAGCTTCTGAACTTGAAGATACTTTAggtgg                                                                                                                                                                                         <  1:164414/301‑1 (MQ=255)`  
                                                                                                                         `gTCCATGATTAGCTCTAAGGTTATCGGCTTAGTGAATCCTGCTGGTATCACCCAGCCACGCCGACTGACCAAAGCTCAGACTGGTGACTTCGTTACTGGTCGTCCAGAAGACATCTCGTTCCTCCAACTGGAGAAGCAAGCATACTTTACTGTAGCTAACGCCGTAAGTGACGCTATCGCGGCTCGCCTTTCGTTTGCCTTTATGTTGAACTCTGCGGTTCAGCGTACAGGTGAACGTGTGACCGCCGAAGAGATTCGGTATGTAGCTTCTGAACTTGAAGATACTTTAGGTGGCTGTCtc                                                                                                                                                                                  >  1:237983/1‑300 (MQ=255)`  
                                                                                                                         `gTCCATGATTAGCTCTAAGGTTATCGGCTTAGTGAATCCTGCTGGTATCACCCAGCCACGCCGACTGACCAAAGCTCAGACTGGTGACTTCGTTACTGGTCGTCCAGAAGACATCTCGTTCCTCCAACTGGAGAAGCAAGCAGACTTTACTGTAGCTAACGCCGTAAGTGACGCTATCGCGGCTCGCCTTTCGTTTGCCTTTATGTTGAACTCTTCGGTTCAGCGTACAGGTGAACGTGTGACCGCCGACGAGATTCGGTATTTAGCTTCTGAACTTGAAGATACTTTAGGTGG‑TGTCTAc                                                                                                                                                                                 >  1:81297/1‑301 (MQ=255)`  
                                                                                                                         `gTCCATGATTAGCTCTAAGGTTATCGGCTTAGTGAATCCTGCTGGTATCACCCAGCCACGCCGACTGACCAAAGCTCAGACTGGTGACTTCGTTACTGGTCGTCCAGAAGACATCTCGTTCCTCCAACTGGAGAAGCAAGCAGACTTTACTGTAGCTAACGCCGTAAGTGACGCTATCGCGGCTCGCCTTTCGTTTGCCTTTATGTTGAACTCTGCGGTTCAGCGTACAGGTGAACGTGTGACCGCCGAAGAGATTCGGTATGTAGCTTCTGAACTTGAAGATACTTTAGGTGG‑TGTCTAc                                                                                                                                                                                 >  1:84606/1‑301 (MQ=255)`  
                                                                                                                          `tCCATGATTAGCTCTAAGGTTATCGGCTTAGTGAATCCTGCTGGTATCACCCAGCCACGCCGACTGACCAAAGCTCAGACTGGTGACTTCGTTACTGGTCGTCCAGAAGACATCTCGTTCCTCCAACTGGAGAAGCAAGCAGACTTTACTGTAGCTAACGCCGTAAGTGACGCTATCGCGGCTCGCCTTTCGTTTGCCTTTATGTTGAACTCTGCGGTTCAGCGTACAGGTGAACGTGTGACCGCCGAAGAGATTCGGTATGTAGCTTCTGAACTTGAAGATACTTTAGGTGG‑TGTCTAct                                                                                                                                                                                <  1:75673/301‑1 (MQ=255)`  
                                                                                                                              `tGATTAGCTCTAAGGTTATCGGCTTAGTGAATCCTGCTGGTATCACCCAGCCACGCCGACTGACCAAAGCTCAGACTGGTGACTTCGTTACTGGTCGTCCAGAAGACATCTCGTTCCTCCAACTGGAGAAGCAAGCAGACTTTACTGTAGCTAACGCCGTAAGGGACGCTATCGCGGCTCGCCTTTCGTTTGCCTTTATGTTGAACTCTGCGGTTCAGCGTACAGGTGAACGTGTGACCGCCGAAGAGATTCGGTATGTAGCTTCTGAACTTGAAGATACTTTAGGTGG‑TGTCTACTCTAt                                                                                                                                                                            <  1:148583/301‑1 (MQ=255)`  
                                                                                                                               `gATTAGCTCTAAGGTTATCGGCTTAGTGAATCCTGCTGGTATCACCCAGCCACGCCGACTGACCAAAGCTCAGACTGGTGACTTCGTTACTGGTCGTCCAGAAGACATCTCGTTCCTCCAACTGGAGAAGCAAGCAGACTTTACTGTAGCTAACGCCGTAAGTGACGCTATCGCGGCTCGCCTTTCGTTTGCCTTTATGTTGAACTCTGCGGTTCAGCGTACAGGTGAACGTGTGACCGCCGAAGAGATTCGGTATGTAGCTTCTGAACTTGAAGATAcctgtctcttatacacatctccg                                                                                                                                                                            >  1:246155/1‑279 (MQ=255)`  
                                                                                                                               `gATTAGCTCTAAGGTTATCGGCTTAGTGAATCCTGCTGGTATCACCCAGCCACGCCGACTGACCAAAGCTCAGACTGGTGACTTCGTTACTGGTCGTCCAGAAGACATCTCGTTCCTCCAACTGGAGAAGCAAGCAGACTTTACTGTAGCTAACGCCGTAAGTGACGCTATCGCGGCTCGCCTTTCGTTTGCCTTTATGTTGAACTCTGCGGTTCAGCGTACAGGTGAACGTGTGACCGCCGAAGAGATTCGGTATGTAGCTTCTGAACTTGAAGATACTTTAGGTGG‑TGTCTACTCTATc                                                                                                                                                                           >  1:42239/1‑301 (MQ=255)`  
                                                                                                                                `aTTAGCTGTAAGGTTAGCGGCTTAGTGAATCCTGCTGGTATCACCCAGCCACGCCGACTGACCAAAGCTCAGACTGGTGACTTCGGTACTGGTCGTCCAGAAGACATCTCGTTCCTCCAACTGGAGAAGCAAGCAGACTTTACTGTAGCTAACGCCGTAAGTGACGCTATCGCGGCTCGCCTTTCGTTTGCCTTTATGTTGAACTCTGCGGTTCAGCGTACAGGTGAACGTGTGACCGCCGAAGAGATTCGGTATGTAGCTTCTGAACTTGAAGATACTTTAGGTGG‑TGTCTACTCTATcc                                                                                                                                                                          <  1:250163/301‑1 (MQ=255)`  
                                                                                                                                `aTTAGCTCTAAGGTTATCGGCTTAGTGAATCCTGCTGGTATCACCCAGCCACGCCGACTGACCAAAGCTCAGACTGGTGACTTCGTTACTGGTCGTCCAGAAGACATCTCGTTCCTCCAACTGGAGAAGCAAGCAGACTTTACTGTAGCTAACGCCGTAAGTGACGCTATTGCGGCTCGCCTTTCGTTTGCCTTTATGTTGAACTCTGCGGTTCAGCGTACAGGTGAACGTGTGACCGCCGAAGAGATTCGGTATGTAGCTTCTGAACTTGAAGATACTTTAGGTGG‑TGTCTACTCTATcc                                                                                                                                                                          <  1:168131/301‑1 (MQ=255)`  
                                                                                                                                `aTTAGCTCTAAGGTTATCGGCTTAGTGAATCCTGCTGGTATCACCCAGCCACGCCGACTGACCAAAGCTCAGACTGGTGACTTCGTTACTGGTCGTCCAGAAGACATCTCGTTCCTCCAACTGGAGAAGCAAGCAGACTTTACTGTAGCTAACGCCGTAAGTGACGCTATCGCGGCTCGCCTTTCGTTTGCCTTTATGTTGAACTCTGCGGTTCAGCGTACAGGTGAACGTGTGACCGCCGAAGAGATTCGGTATGTAGCTTCTGAACTTGAAGATACTTTAGGTGG‑TGTCTACTCTATcc                                                                                                                                                                          <  1:321892/301‑1 (MQ=255)`  
                                                                                                                                `aTTAGCTCTAAGGTTATCGGCTTAGTGAAGCCTGCTGGTATCACCCAGCCACGCCGACTGACCAAAGCTCAGACTGGTGACTTCGTTACTGGTCGTCCAGAAGACATCTCGTTCCTCCAACTGGAGAAGCAAGCAGACTTTACTGTAGCTAACGCCGTAAGTGACGCTATCGAGGCTCGCCTTTCGTTTGCCTTTATGTTGAACTCTGCGGTTCAGCGTACAGGTGAACGTGTGACCGCCGAAGAGATTCGGTATGTAGCTTCTGAACTTGAAGATACTTTAGGTGG‑TGTCTACTCTATcc                                                                                                                                                                          <  1:270158/301‑1 (MQ=255)`  
                                                                                                                                `aTTAGCTATAAGGTTATCGGCTTAGTGAATCCTGCTGGTATCACCCAGCAACGACGAATGACCAAAGCTCAGACTGGTGACTTCGTTACTGGTCGTCCAGAAGACATCTCGTTCCTCCAACTGGAGAAGCAAGCAGAAGTTACTGTAGCTAACGCCGTAAGTGACGATATCGCGGCTGGCCTTTCGTTTGCCGTTATGTTGAACTCTGAGGTTCAGCGTACAGGTGAACGTGTGACCGCCGAAGAGATTCGGTATGTAGCTTCTGAACTTGAAGATACTTTAGGTGG‑GGTCTACTCTATcc                                                                                                                                                                          <  1:38704/301‑1 (MQ=255)`  
                                                                                                                                    `gCTCTAAGGTTATCGGCTTAGTGAATCCTGCTGGTATCACCCAGCCACGCCGACTGACCAAAGCTCAGACTGGTGACTTCGTTACTGGTCGTCCAGAAGACATCTCGTTCCTCCAACTGGAGAAGCAAGCAGACTTTACTGTAGCTAACGCCGTAAGTGACGCTATCGCGGCTCGCCTTTCGTTTGCCTTTATGTTTAACTCTTCGGTTCAGCTTCCAGTTGAACGTGTGCCCGCCGAAGCGATTCGGTATGTTGCTTCTGACCTTGAAGATACTTTATGTTG‑TGTCTtctcctgctcctt                                                                                                                                                                      >  1:318096/1‑292 (MQ=255)`  
                                                                                                                                    `gCTCTAAGGTTATCGGCTTAGTGAATCCTGCTGGTATCACCCAGCCACGCCGACTGACCAAAGCTCAGACTGGTGACTTCGTTACTGGTCGTCCAGAAGACATCTCGTTCCTCCAACTGGAGAAGCAAGCAGACTTTACTGTAGCTAACGCCGTAAGTGACGCTATCGCGGCTCGCCTTTCGTTTGCCTTTATGTTGAACTCTGCGGTTCAGCGTACAGGTGAACGTGTGACCGCCGAAGAGATTCGGTATGTCGCTTCTGAACTTTAAGATACTTTAGGTGG‑TGTCTACTCTATCCtttt                                                                                                                                                                      >  1:126416/1‑300 (MQ=255)`  
                                                                                                                                            `gTTATCGGCTTAGTGAATCCTGCTGGTATCACCCAGCCACGCCGACTGACCAAAGCTCAGACTGGTGACTTCGTTACTGGTCGTCCAGAAGACATCTCGTTCCTCCAACTGGAGAAGCAAGCAGACTTTACTGTAGCTAACGCCGTAAGTGACGCTATCGCGGCTCGCCTTTCGTTTGCCTTTATGTTGAACTCTGCGGTTCAGCGTACAGGTGAACGTGTGACCGCCGAAGAGATTCGGTATGTAGCTTCTGCACTTGAAGATACTTTAGGTGG‑TTTCTACTCTATCCTTTCTCCAGAAt                                                                                                                                                              >  1:145459/1‑301 (MQ=255)`  
                                                                                                                                                 `cGGCTTAGTGAATCCTGCTGGTATCACCCAGCCACGCCGACTGACCAAAGCTCAGACTGGTGACTTCGTTACTGGTCGTCCAGAAGACATCTCGTTCCTCCAACTGGAGAAGCAAGCAGACTTTACTGTAGCTAACGCCGTAAGTGACGCTATCGAGGCTCGCCTTTCGTTTGCCTTTATGTTGAACTCTGCGGTTCAGCGTACAGGTGAACGTGTGACCGCCGAAGAGATTCGGTATGTAGCTTCTGAACTTGAAGATACTTTAGGTGG‑TGTCTACTCTATCCTTTCTCAAGAATTACaa                                                                                                                                                         <  1:134964/301‑1 (MQ=255)`  
                                                                                                                                                  `ggCTTAGTGAATCCTGCTGGTATCACCCAGCCACGCCGACTGACCAAAGCTCAGACTGGTGACTTCGTTACTGGTTGTCCAGAAGACATCTCGTTCCTCCAACTGGAGAAGCAAGCAGACTTTACTGTAGCTAACGCCGTAAGTGACGCTATCGCGGCTCGCCTTTCGTTTGCCTTTATGTTGAACTCTGCGGTTCAGCGTACAGGTGAACGTGTGACCGCCGAAGAGATTCGGTATGTAGCTTCTGAACTTGAAGATACTTTAGGTGG‑TGTCTACTCTATCCTTTCTCAAGAATTACAAt                                                                                                                                                        >  1:307764/1‑301 (MQ=255)`  
                                                                                                                                                  `ggCTTAGTGAATCCTGCTGGTATCACCCAGCCACGCCGACTGACCAAAGCTCAGACTGGTGACTTCGTTACTGGTCGTCCAGAAGACATCTCGTTCCTCCAACTGGAGAAGCAAGCAGACTTTACTGTAGCTAACGCCGTAAGTGACGCTATCGCGGCTCGCCTTTCGTTTGCCTTTATGTTGAACTCTGCGGTTCAGCGTACAGGTGAACGTGTGACCGCCGAAGAGATTCGGTATGTAGCTTCTGAACTTGAAGATACTTTAGGTGG‑TGTCTACTCTATCCTTTCTCAAGAATTAcctt                                                                                                                                                        >  1:187904/1‑298 (MQ=255)`  
                                                                                                                                                            `aTCCTGCTGGTATCACCCAGCCACGCCGACTGACCAAAGCTCAGACTGGTGACTTCGTTACTGGTCGTCCAGAAGACATCTCGTTCCTCCAACTGGAGAAGCAAGCAGACTTTACTGTAGCTAACGCCGTAAGTGACGCTATCGCGGCTCGCCTCTCGTTTGCCTTTATGTTGAACTCTGCGGTTCAGCGTACAGGTGAACGTGTGACCGCCGAAGAGATTCGGTATGTAGCTTCTGAACTTGAAGATACTTTAGGTGG‑TGTCTACTCTATCCTTTCTCAAGAATTAAAATTGCCTCTGGt                                                                                                                                              >  1:345590/1‑301 (MQ=255)`  
                                                                                                                                                              `ccTGCTGGTATCACCCAGCCACGCCGACTGACCAAAGCTCAGACTGGTGACTTCGTTACTGGTAGTCCAGAAGACATCTCGTTCCTCCAACTGGAGAAGCAAGCAGACTTTACTGTAGCTAACGCCGTAAGTGACGCTATCGCGGCTCGCCTTTCGATTGCCTTTATGTTGAACTCTTCGGTTCAGCGTACAGGTGAACGTGTGACCGCCGAAGAGATTCGGTATGTAGCTTCTGAACTTGAAGATACTTTAGGTGG‑TGTCTACTCTATCCTTTCTCAAGAATTACAATTGCCTCTGGTAc                                                                                                                                            >  1:219327/1‑301 (MQ=255)`  
                                                                                                                                                               `ctgctgGTATCACCCAGCCACGCCGACTGACCAAAGCTCAGACTGGTGACTTCGTTACTGGTCGTCCAGAAGACATCTCGTTCCTCCAACTGGAGAAGCAAGCAGACTTTACTGTAGCTAACGCCGTAAGTGACGCTATCGCGGCTCGCCTTTCGTTTGCCTTGATGTTGAACTCTGCGGTTCAGCGTACAGGTGAACGTGGGACCGCCGAAGAGATTCGGGATGTAGCTTCGGAACTTGAGGATACTTTAGGGGG‑TGTCTACTCTATCCTTTCTCAAGAATTACAATTGccgcgtgtccg                                                                                                                                           >  1:304515/1‑292 (MQ=255)`  
                                                                                                                                                               `ctgctgGTATCACACAGCCACGCCGACTGACCAAAGCTCAGACTGGTTACTTCGTTACTGGTCGTCCAGAAGACATCTCGTTCCTCCAACTGGAGAAGCAAGCAGACTTTACTGTAGCTAACGCCGTAAGTGACGCTATCGCGGCTCGCCTTTCGTTTGCCTTTATGTTGAACTCTGCGGTTCAGCGTACAGGTGAACGTGTGACCGCCGAAGAGATTCGGTATGTAGCTTCTGAACTTGAAGATACTTTCGGTGG‑TGTCTACTCCATCCTTTCTCACGAATTACCAtttctgtttctttt                                                                                                                                           >  1:243216/1‑289 (MQ=255)`  
                                                                                                                                                                              `aGCCACGCCGACTGACCAAAGCTCAGACTGGTGACTTCGTTACTGGTCGTCCAGAAGACATCTCGTTCCTCCAACTGGAGAAGCAAGCAGACTTTACTGTAGCTAACGCCGTAAGTGACGCTATCGCGGCTCGCCTTTCGTTTGCCTTTATGTTGAACTCTGCGGTTCAGCGTACAGGTGAACGTGTGACCGCCGAAGAGATTCGGTATGTAGCTTCTGAACTTGAAGATACTTTAGGTGG‑TGTCTACTCTATCCTTTCTCAAGAATTACAATTGCCTCTGGTACGAGTGCTCTTGAAGCa                                                                                                                            <  1:59590/301‑1 (MQ=255)`  
                                                                                                                                                                              `aGCCACGCCGACTGACCAAAGATCAGACTGGTGACTTCGTTACTGGTCGTCCAGAAGACATCTCGTTCCTCCAACTGGAGAAGCAAGCAGACTTTACTGTAGCTAACGCCGTAAGTGACGCTATCGCGGCTCGCCTTTCGTTTGCCTTTATGTTGAACTATGCGGTTCAGCGTACAGGTGAACGTGTGACCGCCGAAGAGATTCGGTATGTAGCTTCTGAACTTGAAGATACTTTAGGTGG‑TGTCTACTCTATCCTTTCTCAAGAATTACAATTGCCTCTGGTACGAGTGCTCTTGAAGCa                                                                                                                            <  1:152514/301‑1 (MQ=255)`  
                                                                                                                                                                                       `gACTGACCAAAGCTCAGACTGGTGACTTCGTTACTGGTCGTCCAGAAGACATCTCGTTCCTCCAACTGGAGAAGCAAGCAGACTTTACTGTAGCTAACGCCGTAAGTGACGCTATCGCGGCTCGCCTTTCGTTTGCCTTTATGTTGAACTCTGCGGTTCAGCGTACAGGTGAACGTGTGACCGCCGAAGAGATTCGGTATGTAGCTTCTGAACTTGAAGATACTTTAGGTGG‑TGTCTACTCTATCCTTTCTCAAGAATTACAATTGCCTCTGGTACGAGTGCTCTTGAAGCAACTACAAGc                                                                                                                   <  1:48652/301‑1 (MQ=255)`  
                                                                                                                                                                                               `ggagatgtgtataagagacaggTTACTGGTCGTCCAGAAGACATCTCGTTCCTCCAACTGGAGAAGCAAGCAGACTTTACTGTAGCTAACGCCGTAAGTGACGCTATCGCGGCTCGCCTTTCGTTTGCCTTTATGTTGAACTCTGCGGTTCAGAGTACAGGTGAACGTGTGACCGCCGAAGAGATTCGGTATGTAGCTTCTGAACTTGAAGATACTTTAGGTGG‑TGTCTACTCTATCCTTTCTCAAGAATTACAATTGCCTCTGGTACGAGTGCTCTTGAAGCAACTACAAGCCACGCAAc                                                                                                           <  1:211663/280‑1 (MQ=255)`  
                                                                                                                                                                                                `aaGCTCAGACTGGTGACTTCGTTACTGGTCGTCCAGAAGACATCTCGTTCCTCCAACTGGAGAAGCAAGCAGACTTTACTGTAGCTAACGCCGTAAGTGACGCTATCGCGGCTCGCCTTTCGTTTGCCTTTATGTTGAACTCTGCGGTTCAGCGTACAGGTGAACGTGTGACCGCCGAAGAGATTCGGTATGTAGCTTCTGAACTTGAAGATACTTTAGGTGG‑TGTCTACTCTATCCTTTCTCAAGAATTACAATTGCCTCTGGTACTAGTGCTCTTGAAGCAACTACAAGCCACGCAACa                                                                                                          >  1:334668/1‑301 (MQ=255)`  
                                                                                                                                                                                                     `cAGACTGGTGACTTCGTTACTGGTCGTCCAGAAGACATCTCGTTCCTCCAACTGGAGAAGCAAGCAGACTTTACTGTAGCTAACGCCGTAAGTGACGCTATCGCGGCTCGCCTTTCGTTTGCCTTTATGTTGAACTCTGTGGTTCAGCGTACAGGTGAACGTGTGACCGCCGAAGAGATTCGGTATGTAGCTTCTGAACTTGAAGATACTTTAGGTGG‑TGTCTACTCTATCCTTTCTCAAGAATTACAATTGCCTCTGGTACGAGTGCTCTTGAAGCAACTACAAGCCACGCAACAGATTc                                                                                                     <  1:10021/301‑1 (MQ=255)`  
                                                                                                                                                                                                      `aGACTGGTGCCTTCGTTACTGGTCGTCCAGAAGACATCTCGTTCCTCCAACTGGAGAAGCAAGCAGACTTTACTGTAGCTAACGCCGTAAGTGACGCTATCGAGGCTCGCCTTTCGTTTGCCATTATGTTTAACTCTGCGGTTCAGCGTACAGGTGAACGTGTGACCGCCGAAGAGATTCGGTATGTAGCTTCTGAACTTGAAGATACTTTAGGTGG‑TGTCTACTCTATCCTTTCTCAAGAATTACAATTGCCTCTGGTACGAGTGCTCTTGAAGCAACTACAAGCCACGCAACAGATTcc                                                                                                    <  1:202278/301‑1 (MQ=255)`  
                                                                                                                                                                                                          `gctcggagatgtgtataagagacaggaagaCATCTCGTTCCTCCAACTGGAGAAGCAAGCAGACTTTACTGTAGCTAACGCCGTAAGTGACGCTATCGCGGCTCGCCTTTCGTTTGCCTTTATGTTGAACTCTGCGGTTCAGCGTACAGGTGAACGTGTGACCGCCGAAGAGATTCTGTATGTAGCTTCTGAACTTGAAGATACTTTAGGTGG‑TGTCTACTCTATCCTTTCTCAAGAATTACAATTGCCTCTGGTACGAGTGCTCTTGAAGCAACTACAAGCCACGCAACAGATTCCTGAg                                                                                                <  1:157379/276‑1 (MQ=255)`  
                                                                                                                                                                                                                     `ttACTGGTCGTCCAGAAGACATCTCGTTCCTCCAACTGGAGAAGCAAGCAGACTTTACTGTAGCTAACGCCGTAAGTGACGCTATCGCGGCTCGCCTTTCGTTTGCCTTTATGTTGAACTCTGCGGTTCAGCGTACAGGTGAACGTGTGACCGCCGAAGAGATTCGGTATGTAGCTTCTGAACTTGAAGATACTTTAGGTGG‑TGTCTACTCTATCCTTTCTCAAGAATTACAATTGCCTCTGGTACGAGTGCTCTTGAAGCAACTACAAGCCACGCAACAGATTCCTGAGTTACCTAAGGa                                                                                     <  1:273551/301‑1 (MQ=255)`  
                                                                                                                                                                                                                     `aagggacaggtcCAGAAGACATCTCGTTCCTCCAACTGGAGAAGCAAGCAGACTTTACTGTAGCTAACGCCGTAAGTGAAGCTATCGCGGCTCGCCTTTCGTTTGCCTTTATGTTGAACTCTGCGGTTCAGCGTACAGGTGAACGTGTGACCGCCGAAGAGATTCGGTATGTAGCTTCTGAACTTGAAGATACTTTAGGTGG‑TGTCTACTCTATCCTTTCTCAAGAATTACAATTGCCTCTGGTACGAGTGCTCTTGAAGCAACTACAAGCCACGCAACAGATTCCTGAGTTACCTAAGGa                                                                                     <  1:294348/292‑1 (MQ=255)`  
                                                                                                                                                                                                                      `gatgtgtataagagacagcATCTCGTTCCTCCAACTGGAGAAGCAAGCAGACTTTACTGTAGCTAACGCCGTAAGTGACGCTATCGCGGCTCGCCTTTCGTTTGCCTTTATGTTGAACTCTGCGGTTCAGCGTACAGGTGAACGTGTGACCGCCGAAGAGATTCGGTATGTAGCTTCTGAACTTGAAGATACTTTAGGTGG‑TGTCTACTCTATCCTTTCTCAAGAATTACAATTGCCTCTGGTACGAGTGCTCTTGAAGCAACTACAAGCCACGCAACAGATTCCTGAGTTACCTAAGGaa                                                                                    <  1:229063/283‑1 (MQ=255)`  
                                                                                                                                                                                                                          `ggTCGTCCAGAAGACATCTCGTTCCTCCAACTGGAGAAGCAAGCAGACTTTACTGTAGCTAACACCGTAAGTGACGCTATCGCGGCTCGCCTTTCGTTTGCCTTTATGTTGAACTCTGCGGTTCAGCGTACAGGTGAACGTGTGACCGCCGAAGAGATTCGGTATGTAGCTTCTGAACTTGAAGATACTTTAGGTGG‑TGTCTACTCTATCCTTTCTCAAGAATTACAATTGCCTCTGGTACGAGTGCTCTTGAAGCAACTACAAGCCACGCAACAGactgtctcttatacacatctccgag                                                                                >  1:129462/1‑277 (MQ=255)`  
                                                                                                                                                                                                                              `gtcCAGAAGACATCTCGTTCCTCCAACTGGAGAAGCAAGCAGACTTTACTGTAGCTAACGCCGTAAGTGACGCTATCGCGGCTCGCCTTTCGTTTGCCTTTATGTTGAACTCTGCGGTTCAGCGTACAGGTGAACGTGTGACCGCCGAAGAGATTCGGTATGTAGCTTCTGAACTTGAAGATACTTTAGGTGG‑TGTCTACTCTATCCTTTCTCAAGAATTACAATTGCCTCTGGTACGAGTGCTCTTGAAGCAACTACAAGCCACGCAACAGATTCCTGAGTTACCTAAGGAAGCCGTaga                                                                            >  1:179367/1‑301 (MQ=255)`  
                                                                                                                                                                                                                               `tcCAGAAGACATCTCGTTCCTCCAACTGGAGAAGCAAGCAGACTTTACTGTAGCTAACGCCGTAAGTGACGCTATCGCGGCTCGCCTTTCGTTTGCCTTTATGTTGAACTCTGCGGTTCAGCGTACAGGTGAACGTGTGACCGCCGCAGAGATTCGGTATGTAGCTTCTGAACTTGAAGATACTTTAGGTGG‑TGTCTACTCTATCCTTTCTCAAGAATTACAATTGCCTCTGGTACGAGTGCTCTTGAAGCAACTACGAGCCACGCAACAGATTCCTGAGTTACCTAAGGaactgtctctt                                                                           >  1:327505/1‑292 (MQ=255)`  
                                                                                                                                                                                                                               `tcCAGAAGACATCTCGTTCCTCCAACTGGAGAAGCAAGCAGACTTTACTGTAGCTAACGCCGTAAGTGACGCTATCGCGGCTCGCCTTTCGTTTGCCTTTATGTTGAACTCTGCGGTTCAGCGTACAGGTGAACGTGTGACCGCCGAAGAGATTCGGTATGTAGCTTCTGAACTTGAAGATACTTTAGGTGG‑TGTCTACTCTATCCTTTCTCAAGAATTACAATTGCCTCTGGTACGAGTGCTCTTGAAGCAACTACAAGCCACGCAACAGATTCCTGAGTTACCTAAGGAAGCCGTagag                                                                           <  1:17542/301‑1 (MQ=255)`  
                                                                                                                                                                                                                                `ccAGAAGACATCTCGTTCCTCCAACTGTAGAAGCAAGCAGACTTTACTGTAGCTAACGCCGTAAGTGACGCTATCGCGGCTCGCCTTTCGTTTGCCTTTATGTTGAACTCTGCGGTTCAGCGTACAGGTGAACGTGTGACCGCCGAAGAGATTCGGTATGTAGCTTCTGAACTTGAAGATACTTTAGGTGG‑TGTCTACTCTATCCTTTCTCAAGAATTACAATTGCCTCTGGTACGAGTGCTCTTGAAGCAACTACAAGCCACGCAACAGATTCCTGAGTTACCTAAGGAAGCCGTAGAGc                                                                          <  1:54591/301‑1 (MQ=255)`  
                                                                                                                                                                                                                                   `gaagaCATCTCGTTCCTCCAACTGGAGAAGCAAGCAGACTTTACTGTAGCTAACGCCGTAAGTGACGCTATCGCGGCTCGCCTTTCGTTTGCCTTTATGTTGAACTCTGCGGTTCAGCGTACAGGTGAACGTGTGACCGCCGAAGAGATTCGGTATGTAGCTTCTGAACTTGAAGATACTTTAGGTGG‑TGTCTACTCTATCCTTTCTCAAGAATTACAATTGCCTCTGGTACGAGTGCTCTTGAAGCAACTACAAGCCACGCAACAGATTCCTGAGTTACCTAAGGAAGCCGTAGAGCCaa                                                                       >  1:122638/1‑301 (MQ=255)`  
                                                                                                                                                                                                                                     `agaCATCTCGTTCCTCCAACTGGGGAAGCAATCAGACTTTACTGTAGCTAACGCCGTAAGTGACGCTGTCGCGGCTCGCCTTTCGTTTGCCTTTATGTTGAACTCTGCGGTTCAGCGTACAGGTGAACGTGTGACCGCCGTAGAGATTCGGTATGTAGCTTCTGAACTTGAAGATACTTTAGGTGG‑TGTCTACTCTATCCTTTCTCAAGAATTACAATTGCCTCTGGTACGAGTGCTCTTGAAGCAACTACAAGCCACGCAACAGATTCCTGAGTTACCTAAGGAAGCCGTAGAGCCAAcc                                                                     <  1:65049/301‑1 (MQ=255)`  
                                                                                                                                                                                                                                     `agaCATCTCGTTCCTCCAACTGGAGAAGCAAGCAGACTTTACTGTAGCTAACGCCGTAAGTGACGCTATCGCGGCTCGCCTTTCGTTTGCCTTTATGTTGAACTCTGCGGTTCAGCGGACAGGTGAACGTGTGACCGCCGAAGAGATTCGGTATGTAGCTTCTGAACTTGAAGATACTTTAGGTGG‑TGTCTACTCTATCCTTTCTCAAGAATTACAATTGCCTCTGGTACGAGTGCTCTTGAAGCAACTACAAGCCACGCAACAGATTCCTGAGTTACCTAAGGAAGCCGTAGAGCCAAcc                                                                     <  1:199426/301‑1 (MQ=255)`  
                                                                                                                                                                                                                                      `gaCATCTCGTTCCTCCAACTGGAGAAGCAAGCAGACTTTACTGTAGCTAACGCCGTAAGTGACGCTATCGAGGCTCGCCTTTCGTTTGCCTTTATGTTGAACTCTGCGGTTCAGCGTACAGGTGAACGTGTGACCGCCGAAGAGATTCGGTATGTAGCTTCTGAACTTGAAGATACTTTAGGTGG‑TGTCTACTCTATCCTTTCTCAAGAATTACAATTGCCTCTGGTACGAGTGCTCTTGAAGCAACTACAAGCCACGCAACAGATTCCTGAGTTACCTACGGAAGCCgctgtctcttata                                                                    >  1:193102/1‑289 (MQ=255)`  
                                                                                                                                                                                                                                             `ggatcggagatgggtataagagacaggACTTTACTGTAGCTAACGCCGTAAGTGACGCTATCGCGGATCGCCTTTTGTTTGCCTTTATGTTGAACTCTGCGGTTCAGCGTACAGGTGAACGTGTGGCCGCCGAAGAGATTCGGTATGTAGCTTCTGAACTTGAAGATACTTTAGGTGG‑TGTCTACTCTATCCTTTCTCAAGAATTACAATTGCCTCTGGTACGAGTGCTCTTGAAGCAACTACAAGCCACGCAACAGATTCCTGAGTTACCTAAGGAAGCCGTAGAGCCAACCATTAGTAc                                                             <  1:276745/275‑1 (MQ=255)`  
                                                                                                                                                                                                                                             `cGTTCCTCCAACTGGAGAAGCAAGCAGACTTTACTGTAGCTAACGCCGTAAGTGACGCTATCGCGGCTCGCCTTTCGTTTGCCTTTATGTTGAACTCTGCGGTTCAGCGTACAGATGAACGTGTGACCGCCGAAGAGATTCGGTATGTAGCTTCTGAACTTGAAGATACTTTAGGTGG‑TGTCTACTCTATCCTTTCTCAAGAATTACAATTGCCTCTGGTACGAGTGCTCTTGAAGCAACTACAAGCCACGCAACAGATTCCTGAGTTACCTAAGGAAGCCGTAGAGCCAACCATTAGTAc                                                             <  1:114359/301‑1 (MQ=255)`  
                                                                                                                                                                                                                                             `acagcctccAACTGGAGAAGCAAGCAGACTTTACTGTAGCTAACGCCGTAAGTGACGCTATCGCGGCTCGCCTTTCGTTTGCCGTTATGTTGAACTCTGCGGTTCAGCGTACAGGTGAACGTGTGACCGCCGAAGAGATTCGGTATGTAGCTTCTGAACTTGAAGATACTTTAGGTGG‑TGTCTACTCTATCCTTTCTCAAGAATTACAATTGCCTCTGGTACGAGTGCTCTTGAAGCAACTACAAGCCACGCAACAGATTCCTGAGTTACCTAAGGAAGCCGTAGAGCCAACCATTAGTAc                                                             <  1:132397/297‑1 (MQ=255)`  
                                                                                                                                                                                                                                                        `cTGGAGAAGCAAGCAGACTTTACTGTAGCTAACGCCGTAAGTGACGCTATCGCGGCTCGCCTGTCGTTTGCCTTTATGTTGAACTCGGCGGTTCAGCGTACAGGTGAACGTGTGACCGCCGAAGAGATTCGGTATGTAGCTTCTGAACTTGAAGATACTTTAGGTGG‑TGTCTACTCTATCCTTTCTCAAGAATTACAATTGCCTCTGGTACGAGTGCTCTTGAAGCAACTACAAGCCACGCAACAGATTCCTGAGTTACCTAAGGAAGCCGTAGAGCCAACCATTAGTACAGGTCTGGAAg                                                  <  1:101997/301‑1 (MQ=255)`  
                                                                                                                                                                                                                                                               `agcaagcaGACTTTACTGTAGCTAACGCCGTAAGTGACGCTATCGCGGCTCGCCTTTCGTTTGCCTTTATGTTGAACTCTGCGGTTCAGCGTACAGGTGAACGTGTGACCGCCGAAGAGATTCGGTATGTAGCTTCTGAACTTGAAGATACTTTAGGTGG‑TGTCTACTCTATCCTTTCTCAAGAATTACAATTGCCTCTGGTACGAGTGCTCTTGAAGCAACTACAAGCCACGCAACAGATTCCTGAGTTACCTAAGGAAGCCGTAGAGCCAACCATTAGTACAGGTCTGGAAGCAATTgg                                           >  1:297350/1‑301 (MQ=255)`  
                                                                                                                                                                                                                                                                      `aGACTTTACTGTAGCTAACGCCGTAAGTGACGCTATCGCGGCTCGCCTTTCGTTTGCCTTTATGTTGAACTCTGCGGTTCAGCGTACAGGTGAACGTGTGACCGCCGAAGAGATTCGGTATGTAGCTTCTGAACTTGAAGATACTTTAGGTGG‑TGTCTACTCTATCCTTTCTCAAGAATTACAATTGCCTCTGGTACGAGTGCTCTTGAAGCAACTACAAGCCACGCAACAGATTCCTGAGTTACCTAAGGAAGCCGTAGAGCCAACCATTAGTACAGGTCTGGAAGCAATTGGTCGAGGa                                    >  1:171581/1‑301 (MQ=255)`  
                                                                                                                                                                                                                                                                       `gACTTTACTGTAGCTAACGCCGTAAGTGACGCTATCGCGGCTCGCCTTTCGTTTGCCTTTATGTTGAACTCTGCGGTTCAGCGTACAGGTGAACGTGTGACCGCCGAAGAGATTCGGTATGTAGCTTCTGAACTTGAAGATACTTTAGGTGG‑TGTCTACTCTATCCTTTCTCAAGAATTACAATTGCCTCTGGTACGAGTGCTCTTGAAGCAACTACAAGCCACGCAACAGATTCCTGAGTTACCTAAGGAAGCCGTAGAGCCAACCATTAGTACAGGTCTGGAAGCAATTGGTCGAGGAc                                   >  1:204802/1‑301 (MQ=255)`  
                                                                                                                                                                                                                                                                         `cTTTACTGTAGCTAACGCCGTAAGTGACGCTATCGCGGCTCGCCTTTCGTTTGCCTTTATGTTGAACTCTGCGGTTCAGCGTACAGGTGAACGTGTGACCGCCGAAGAGATTCGGTATGTAGCTTCTGAACTTGAAGATACTTTAGGTGG‑TGTCTACTCTATCCTTTCTCAAGAATTACAATTGCCTCTGGTACGAGTGCTCTTGAAGCAACTACAAGCCACGCAACAGATTCCTGTGTTACCTAAGGAAGCCGTAGAGCCAACCATTAGTACAGGTCTGGAAGAATTTGGTAGAGGACaa                                 >  1:340669/1‑301 (MQ=255)`  
                                                                                                                                                                                                                                                                           `ttACTGTAGCTAACGCCGTAAGTGACGCTATCGAGGCTCGCCTTTCGTTTGCCTTTATGTTGAACTCTGCGGTTCAGCGTACAGGTGAACGTGTGACCGCCGAAGAGATTCGGTATGTAGCTTCTGAACTTGAAGATACTTTAGGTGG‑TGTCTACTCTATCCTTTCTCAAGAATTACAATTGCCTCTGGTACGAGTGCTCTTGAAGCAACTACAAGCCACGCAACAGATTCCTGAGTTACCTAAGGAAGCCGTAGAGCCAACCATTAGTACAGGTCTGGAAGCAATTGGTCGAGGACAAGa                               >  1:232094/1‑301 (MQ=255)`  
                                                                                                                                                                                                                                                                            `tACTGTAGCTAACGCCGTAAGTGACGCTATCGCGGCTCGCCTTTCGTTTGCCTTTATGTTGAACTCTGCGGTTCAGCGTACAGGTGAACGTGTGACCGCCGAAGAGATTCGGTATGTAGCTTCTGAACTTGAAGATACTTTAGGTGG‑TGTCTACTCTATCCTTTCTCAAGAATTACAATTGCCTCTGGTACGAGTGCTCTTGAAGCAACTACAAGCCACGCAACAGATTCCTGAGTTACCTAAGGAAGCCGTAGAGCCAACCATTAGTACAGGTCTGGAAGCAATTGGTCGAGGACAAGAc                              <  1:89985/301‑1 (MQ=255)`  
                                                                                                                                                                                                                                                                                  `aGCTAACGCCGTAAGTGACGCTATCGCGGCTCGCCTTTCGTTTGCCTTTATGTTGAACTCTGCGGTTCAGCGTACAGGTGAACGTGTGACCGCCGAAGAGATTCGGTATGTAGCTTCTGAACTTGAAGATACTTTAGGTGG‑TGTCTACTCTATCCTTTCTCAAGAATTACAATTGCCTCTGGTACGAGTGCTCTTGAAGCAACTACAAGCCACGCAACAGATTCCTGAGTTACCTAAGGAAGCCGTAGAGCCAACCATTAGTACAGGTCTGGAAGCAATTGGTCGAGGACAAGACCTTGAt                        <  1:216325/301‑1 (MQ=255)`  
                                                                                                                                                                                                                                                                                     `gtgtataagagacagcGCTATCGCGGCTCGACTTTCGTTTGCCTTTATGTTGAACTCTGCGGTTCAGCGTACAGGTGAACGTGTGACCGCCGAAGAGATTCGGTATGTAGCTTCTGAACTTGAAGATACTTTAGGTGG‑TGACTACTCTATCCTTTCTCAAGAATTACAATTGCCTCTGGTACGAGTGCTCTTGAAGCAACTACAAGCCACGCAACAGATTCCTGAGTTACCTAAGGAAGCCGTAGAGCCAACCATTAGTACAGGTCTGGAAGCAATTGGTCGAGGACAAGACCTTGATAAg                     <  1:301234/286‑1 (MQ=255)`  
                                                                                                                                                                                                                                                                                        `cgCCGTAAGTGACGCTATCGCGGCTCGCCTTTCGTTTGCCTTTATGTTGAACTCTGCGGTTCAGCGTACAGGTGAACGTGTGACCGCCGAAGAGATTCGGTATGTAGCTTCTGAACTTGAAGATACTTTAGGTGG‑TGTCTACTCTATCCTTTCTCAAGAATTACAATTGCCTCTGGTACGAGTGCTCTTGAAGCAACTACAAGCCACGCAACAGATTCCTGAGTTACCTAAGGAAGCCGTAGAGCCAACCATTAGTACAGGTCTGGAAGCAATTGGTCGAGGACAAGACCTTGATAAGCTg                  >  1:96702/2‑301 (MQ=255)`  
                                                                                                                                                                                                                                                                                               `aGTGACGCTATCGCGGCTCGCCTTTCGTTTGCCTTTATGTTGAACTCTGCGGTTCAGCGTACAGGTGAACGTGTGACCGCCGAAGAGATTCGGTATGTAGCTTCTGAACTTGAAGATACTTTAGGTGG‑TGTCTACTCTATCCTTTCTCAAGAATTACAATTGCCTCTGGTACGAGTGCTCTTGAAGCAACTACAAGCCACGCAACAGATTCCTGAGTTACCTAAGGAAGCCGTAGAGCCAACCATTAGTACAGGTCTGGAAGCAATTGGTCGAGGACAAGACCTTGATAAGCTGGAGCGgt           >  1:87333/1‑301 (MQ=255)`  
                                                                                                                                                                                                                                                                                                   `aCGCTATCGAGGCTCGCCTTTCGTTTGCCTTTATGTTGAACTCTGCGGTTCAGCGTACAGGTGAACGTGTGACCGCCGAAGAGATTCGGTATGTAGCTTCTGAACTTGAAGATACTTTAGGTGG‑TGTCTACTCTATCCTTTCTCAAGAATTACAATTGCCTCTGGTACGAGTGCTCTTGCAGCAACTACAAGCCACGCAACAGATTCCTGAGTTACCTAAGGAAGCCGTAGAGCCAACCATTAGTACAGGTCTGGAAGCAATTGGTCGAGGACAAGACCTTGATAAGCTGGAGCGgtgtgt       >  1:283096/1‑301 (MQ=255)`  
                                                                                                                                                                                                                                                                                                      `cTATCGCGGCTCGCCTTTCGTTTGCCTTTATGTTGAACTCTGCGGTTCAGCGTACAGGTGAACGTGTGACCGCCGAAGAGATTCGGTATGTAGCTTCTGAACTTGAAGATACTTTAGGTGG‑TGTCTACTCTATCCTTTCTCAAGAATTACAATTGCCTCTGGTACGAGTGCTCTTGAAGCAACTACAAGCCACGCAACAGATTCCTGAGTTACCTAAGGAAGCCGTAGAGCCAACCATTAGTACAGGTCTGGAAGCAATTGGTCGAGGACAAGACCTTGATAAGCTGGAGCGGTGTGTCAc    <  1:117501/301‑1 (MQ=255)`  
                                                                                                                                                                                                                                                                                                        `aTCGCGGCTCGCCTTTCGTTTGCCTTTATGTTGAACTCTGCGGTTCAGCGTACAGGTGAACGTGTGACCGCCGAAGAGATTCGGTATGTAGCTTCTGAACTTGAAGATACTTTAGGTGG‑TGTCTACTCTATCCTTTCTCAAGAATTACAATTGCCTCTGGTACGAGTGCTCTTGAAGCAACTACAAGCCACGCAACAGATTCCTGAGTTACCTAAGGAAGCCGTAGAGCCAACCATTAGTACAGGTCTGGAAGCAATTGGTCGAGGACAAGACCTTGAtctgtctcttatacacatctccg  >  1:324064/1‑279 (MQ=255)`  
                                                                                                                                                                                                                                                                                                        `aTCGCGGCTCGCCTTTCGTTTGCCTTTATGTTGAACTCTGCGGTTCAGCGTACAGGTGAACGTGTGACCGCCGAAGAGATTCGGTATGTAGCTTCTGAACTTGAAGATACTTTAGGTGG‑TGTCTACTCTATCCTTTCTCAAGAATTACAATTGCCTCTGGTACGAGTGCTCTTGAAGCAACTACAAGCCACGCAACAGATTCCTGAGTTACCTAAGGAAGCCGTAGAGCCAACCATTAGTACAGGTCTGGAAGCAATTGGTCGAGGACAAGACCTTGATAAGCTGGAGCGGTGTGTCACTg  >  1:255902/1‑301 (MQ=255)`  

                                                                                                                                                                                                                                                                                                            `|`                                                                                                                                                                                                                                                                                                           
`GCCCATACATCCCGATTCGGATGGTCAGACTAGATGGTGAATCCTACGGTCGTTCGTACATTGAGGAATACTTAGGTGACTTACGGTCCCTTGAAAATCTCCAAGAGGCTATCGTCAAGATGTCCATGATTAGCTCTAAGGTTATCGGCTTAGTGAATCCTGCTGGTATCACCCAGCCACGCCGACTGACCAAAGCTCAGACTGGTGACTTCGTTACTGGTCGTCCAGAAGACATCTCGTTCCTCCAACTGGAGAAGCAAGCAGACTTTACTGTAGCTAAAGCCGTAAGTGACGCTATCGAGGCTCGCCTTTCGTTTGCCTTTATGTTGAACTCTGCGGTTCAGCGTACAGGTGAACGTGTGACCGCCGAAGAGATTCGGTATGTAGCTTCTGAACTTGAAGATACTTTAGGTGG‑TGTCTACTCTATCCTTTCTCAAGAATTACAATTGCCTCTGGTACGAGTGCTCTTGAAGCAACTACAAGCCACGCAACAGATTCCTGAGTTACCTAAGGAAGCCGTAGAGCCAACCATTAGTACAGGTCTGGAAGCAATTGGTCGAGGACAAGACCTTGATAAGCTGGAGCGGTGTGTCACTG  >  NC_001604.1/20985‑21581`

  

Alignment Legend

Aligned base mismatch/match (shaded by quality score): `ATCG/ATCG` `< 3 ≤` `ATCG/ATCG` `< 11 ≤` `ATCG/ATCG` `< 24 ≤` `ATCG/ATCG` `< 35 ≤` `ATCG/ATCG` `< 38 ≤` `ATCG/ATCG`

Unaligned base: `atcg`    Masked matching base: `atcg`    Alignment gap: `‑`    Deleted base: `‑`
